# The presence of non-criteria manifestations negatively affects the prognosis of seronegative antiphospholipid syndrome patients: a multicenter study

**DOI:** 10.1186/s13075-021-02702-9

**Published:** 2022-01-03

**Authors:** Gilberto Pires da Rosa, Bernardo Sousa-Pinto, Ester Ferreira, Olga Araújo, Giuseppe Barilaro, Paulo Bettencourt, Ricard Cervera, Gerard Espinosa

**Affiliations:** 1 Department of Autoimmune Diseases, Hospital Clínic, Institut d’Investigacions Biomèdiques August Pi i Sunyer (IDIBAPS), University of Barcelona, Barcelona, Catalonia Spain; 2 Faculty of Medicine, University of Porto, Porto, Portugal; 3 MEDCIDS – Department of Community Medicine, Information and Health Decision Sciences, Faculty of Medicine, University of Porto, Porto, Portugal; 4 CINTESIS – Center for Health Technology and Services Research, Porto, Portugal; 5 Department of Internal Medicine, Centro Hospitalar Universitário de São João, Porto, Portugal; 6 Department of Internal Medicine, Hospital CUF, Porto, Portugal

**Keywords:** Antiphospholipid syndrome, Antiphospholipid antibodies, Seronegative, Single positive, Non-criteria manifestations

## Abstract

**Background:**

Seronegative antiphospholipid syndrome (SN-APS) is often defined as the presence of APS criteria manifestations, negative antiphospholipid antibodies (aPL), and coexistence of APS non-criteria manifestations. Nevertheless, the impact of these non-criteria features is still unclear. On a different note, the relevance of one single aPL positive determination in patients with APS manifestations is another domain with limited evidence. We aim to compare the course of SN-APS and single-positive aPL (SP-aPL) patients with that of individuals with APS manifestations without non-criteria features/aPL positivity (controls).

**Methods:**

Retrospective analysis of patients with thrombosis/obstetric morbidity assessed in two European hospitals between 2005 and 2020. Patients were divided into SN-APS, SP-aPL, and control groups. Clinical characteristics, comorbidities, and therapies were compared.

**Results:**

A total of 82 patients were included in the SN-APS group, 88 in the SP-aPL group, and 185 in the control group. In Cox regression model, SN-APS displayed more thrombosis recurrence than controls (HR 3.8, 95% CI 2.2–6.5, *p* < 0.001) even when adjusting for the presence of hereditary thrombophilia, systemic lupus erythematosus, or contraceptive hormonal treatment. In SP-aPL, the difference in thrombosis recurrence did not reach statistical significance (*p* = 0.078). Indefinite anticoagulation (*p* < 0.001 and *p* = 0.008, respectively) and vitamin K antagonist (VKA) use (*p* < 0.001 in both cases) were more common in SN-APS/SP-aPL.

**Conclusion:**

SN-APS displayed more thrombosis recurrence, indefinite anticoagulation, and VKA use than controls without non-criteria manifestations. The presence of such features in patients with thrombosis and negative aPL may negatively impact their clinical course.

## Introduction

The classification criteria for definite antiphospholipid syndrome (APS) [1] are currently facing a revision, with new criteria under development [2]. The call for an update derives from different aspects surrounding the disease, including the existence of numerous patients with a suspicion of APS but not classified as such according to the current criteria. These patients are often referred to as “non-criteria” APS, a label that encompasses a wide range of clinical and laboratory presentations [3].

A subgroup of these patients correspond to the so-called seronegative APS (SN-APS), a term initially defined as “patients with migraine, stroke, several previous miscarriages, thrombocytopenia, and *livedo reticularis*, whose antiphospholipid antibodies (aPL) tests are doggedly negative [4].” Other definitions have been presented, and in a previous publication, we address the different descriptions present in the literature [3], with most publications [5–7] including not only the presence of APS manifestations and aPL negativity, but also the presence of non-criteria manifestations (i.e., clinical manifestations fairly prevalent in APS patients but not included in the classification criteria). The latter comprise both non-obstetric (e.g., thrombocytopenia, valvular heart disease, *livedo reticularis*) and obstetric manifestations (e.g., two spontaneous abortions, late pregnancy morbidity) [8]. The relevance of such manifestations in seronegative patients resides in the fact that they represent additional evidence to reinforce a possible APS diagnosis, instead of alternative diagnoses such as a different thrombophilia or an idiopathic event. Nevertheless, either due to the rarity of the disease or the difficulty to identify and categorize these patients, there are no studies addressing the impact of these non-criteria manifestations as a whole in the clinical course and prognosis of patients exhibiting clinical manifestations of APS.

Another cluster of patients which raises doubts in clinical practice are those in whom aPL testing is positive in only one occasion. The classification criteria for definite APS [1] require aPL positivity on two or more occasions at least 12 weeks apart, and state that classification as APS should be avoided if the positive aPL determination and clinical manifestations are separated by less than 12 weeks. The rationale behind these caveats includes the difficulty to exclude a false-positive aPL elevation due to other causes in the setting of one positive determination (e.g., infection, malignancy, or drugs) [9–11] and the possibility of transient aPL elevation during the acute phase of an event [12, 13]. However, despite a systematic review reporting a similar recurrence rate to the general population in individuals with venous thromboembolism or stroke and only one single aPL determination [14], the included primary studies are scarce and outdated and have important methodological limitations, such as still not including anti-beta-2-glycoprotein I (anti-β2GPI) antibodies or inadequate aPL positivity cut-offs [15–18].

For the abovementioned reasons, much is still unknown and uncertain regarding SN-APS and single-positive (SP-aPL) individuals. Our aim was to describe a cohort of both subsets of patients, and to compare them with patients with thrombosis or pregnancy morbidity but lacking other criteria to be included in these groups (i.e., without non-criteria features or aPL positivity). Herewith, we intend to evaluate the impact of the presence of non-criteria manifestations of APS and SP-aPL in patients with manifestations of APS (either thromboembolic events or pregnancy morbidity). A deeper understanding of the influence of these traits could change the current management and therapy of these individuals and contribute to an eventual inclusion of some additional features in the classification criteria for APS.

## Methods

### Study design

#### Patient selection

We retrospectively reviewed the clinical records of patients assessed in the Autoimmune Diseases, Internal Medicine, Thrombophilia and Obstetrics Departments of two tertiary European hospitals—University Hospital Center of São João (Oporto, Portugal) and Hospital Clínic of Barcelona (Barcelona, Catalonia, Spain)—between January 2005 and December 2020, selecting all those with thrombosis and/or obstetric morbidity fulfilling the APS clinical classification criteria [1], but not fulfilling laboratory criteria. Patients with a major risk factor for a thrombotic event (e.g., recent major surgery, bone fracture, cancer) were excluded. Patients taking oral contraceptive pill (OCP) were excluded if the medication had been started less than 1 year previously to the event. Patients were then divided into the three following groups:

- *Seronegative APS* (using the definition of the nomenclature recently proposed by our research team for non-criteria APS) [3]: patients with thrombosis or obstetric morbidity fulfilling APS classification criteria [1], plus the presence of “non-criteria” manifestations [at least (i) one obstetric, (ii) one *major* non-obstetric, or (iii) two *minor* non-obstetric manifestations—see Table 1], with persistently negative aPL, and exclusion of other thrombophilias. However, the presence of hereditary thrombophilias (i.e., factor V Leiden mutation, prothrombin G20210A mutation, protein C, protein S, or antithrombin deficiency) was accepted if judged as not justifying the whole clinical presentation of the patient.
- *Single-positive aPL (SP-aPL) group*: patients with thrombosis or obstetric morbidity fulfilling APS classification criteria [1] with only one single positive aPL result (regardless of occurring during or outside the acute phase of the event). Patients were excluded if an evident cause for the positivity was identified, such as infection, malignancy, or drugs.
- *Control group*: patients with thrombosis or obstetric morbidity fulfilling APS classification criteria [1], with persistently negative aPL, without non-criteria manifestations fulfilling the criteria for seronegative APS. The presence of hereditary thrombophilias (i.e., factor V Leiden mutation, prothrombin G20210A mutation, protein C, protein S, or antithrombin deficiency) was accepted in order to establish an adequate parallelism with the SN-APS group.

**Table 1:** Included “non-criteria” manifestations of APS [adapted from [3]]

**Non-obstetric manifestations**		**Obstetric**
**Major**^**a**^		Infertility
Acute ischemic encephalopathy	Adrenal hemorrhage	Late IUGR (after 34 weeks)
APS nephropathy	Cardiac microvascular disease	Late pre-eclampsia (after 34 weeks)
Chorea	Evans syndrome	Placental abruption
*Livedo reticularis/racemosa*	Livedoid vasculopathy	Placental hematoma
Longitudinal myelitis	Pulmonary hemorrhage	Preterm birth (>34 to <37 weeks)
Superficial vein thrombosis	Thrombocytopenia	Puerperal pre-eclampsia
Valvular heart disease		Two or more unexplained in vitro fertilization failures
**Minor**^**a**^		Two unexplained spontaneous abortions <10 weeks
*Amaurosis fugax*	Brain MRI white matter lesions	
Cognitive dysfunction	Coombs’ test positivity	
Hemolytic anemia	Ischemic necrosis of bone	
Migraine	Pseudo-multiple sclerosis	
Pulmonary hypertension	Raynaud’s phenomenon	
Seizures	Sensorineural hearing loss	
Splinter hemorrhages		

*APS* Antiphospholipid syndrome, *IUGR* Intrauterine growth restriction

^a^We considered as *major* manifestations those *suggested, recommended, or strongly recommended* to be included as part of the APS criteria revision in the report of the *14th International Congress on Antiphospholipid Antibodies Technical Task Force on APS Clinical Features* [7] and those occurring in higher frequency in the cases categorized as “highly likely APS” in Phase III of the *Development of New International Classification Criteria for Antiphospholipid Syndrome* [19]

Our aim was to establish a comparison between the SP-aPL and SN-APS groups and the control group regarding demographic and clinical characteristics, namely recurrence of events. The study received approval from the Hospital Clínic Ethics Committee (HCB/2020/1259).

### Data collection and definition of variables

We collected information from each patient on demographic data, type of clinical manifestations (thrombotic, obstetric, or both), specific clinical manifestations [thrombosis: arterial, venous, or both; stroke, transient ischemic accident (TIA), acute myocardial infarction, limb ischemia, deep vein thrombosis (DVT), pulmonary embolism (PE), cerebral vein thrombosis (CVT), and retinal vessels thrombosis; obstetric: one or more unexplained deaths of a morphologically normal fetus at or beyond the 10th week of gestation, one or more premature births of a morphologically normal neonate before the 34th week of gestation, three or more unexplained consecutive spontaneous abortions before the 10th week of gestation, placental ischemia]; recurrence of events; number of thrombotic events; number of spontaneous abortions; presence and type of “non-criteria” manifestations (in the case of SN-APS); aPL positivity and profile (in the case of the SP-aPL group); associated autoimmune diseases (AID); presence of autoantibodies [antinuclear antibodies (ANA), anti-double stranded DNA antibodies (anti-dsDNA)] and complement consumption; cardiovascular risk factors (arterial hypertension, dyslipidemia, hyperuricemia, obesity, smoking); and presence of risk factors for thrombosis (OCP and hereditary thrombophilia: factor V Leiden mutation, prothrombin G20210A mutation, protein C, protein S, or antithrombin deficiency). A possible future progression towards systemic lupus erythematosus (SLE), based on clinical manifestations and autoantibodies’ profile, was also noted.

In addition, we collected information on the treatments each patient was under, including indefinite therapy with oral anticoagulation [vitamin K antagonists (VKA) or direct oral anticoagulants (DOAC)], antiplatelet agents or hydroxychloroquine, anticoagulation duration, and recurrence under treatment. In the case of obstetric manifestations, treatment of at least one pregnancy with low-dose aspirin (LDA) monotherapy, low molecular weight heparin (LMWH) monotherapy, LDA/LMWH combination, or hydroxychloroquine was noted.

### Statistical analysis

Continuous variables were described by means and standard deviations or by medians and interquartile ranges (IQR), while categorical variables were described using absolute and relative frequencies. We built univariable logistic regression models to compare SN-APS patients versus controls and SP-aPL patients versus controls. Tested independent variables included clinical manifestations, recurrence, comorbidities, and treatment options. Multivariable analyses were performed when adjustment for confounders was considered required. Seronegative APS patients and SP-aPL patients were also compared against controls regarding the development of thrombosis recurrence—we built univariable and multivariable Cox regression models (adjusting for the presence of hereditary thrombophilia, SLE and OCP—we could not adjust for additional factors due to sample size limitations), considering the time between the first thrombotic event until the development of a second thrombotic event (or, if such event did not occur, the date of the last registered outpatient visit). In addition, Kaplan-Meier curves were obtained.

Exponentials of logistic regression coefficients were interpreted as odds ratio (OR) and exponentials of Cox regression coefficients were interpreted as hazard ratios (HR). Exponentials of regression coefficients were calculated along with their respective 95% confidence intervals (CI). Values of *p* < 0.05 were considered statistically significant. Data were analyzed using SPSS Statistics 26.0 (IBM Corp, Armonk, NY).

## Results

### Patient characteristics

Three hundred and fifty-five patients were included in the analysis: 82 in the SN-APS group, 88 in the SP-aPL group, and 185 in the control group (161 without and 24 with hereditary thrombophilia). Patient characteristics and demographic data are summarized in Table 2, and the non-criteria manifestations of the SN-APS group in Table 3. Groups displayed no significant difference in age (*p* = 0.519) and age of first event (*p* = 0.241). In univariable regression analyses, a significantly lower frequency of males was observed in the SN-APS group in comparison to the control group (OR = 0.5, 95% CI = 0.3–0.9, *p* = 0.022), with no difference (*p* = 0.120) when adjusting for the presence of obstetric patients in each group; on the other hand, no significant differences on gender distribution were found between the SP-aPL and control groups (*p* = 0.107). Complete results are available in Table 2.

**Table 2:** Patient characteristics, demographic data, and comorbidities

Patient group	Control group (***n*** = 185)	Seronegative APS (***n*** = 82)	***P***-value (SN-APS vs. controls)	Single-positive aPL (***n*** = 88)	***P***-value (SP-APS vs. controls)
**Sex (female) (*n*, %)**	123 (66.5)	66 (80.5)	*p* = 0.022	67 (76.1)	*p* = 0.107
**Age (median, IQR)**	45.0 (38.0–50.0)	45.5 (37.0–53.3)	*p* = 0.105	44 (39.0–51.0)	*p* = 0.519
**Age at first event (median, IQR)**	37.5 (29.0–43.0)	35 (30.0–40.4)	*p* = 0.609	35 (27.0–43.0)	*p* = 0.241
**Type of manifestations**					
Thrombosis only (*n*, %)	166 (89.7)	61 (74.4)	*p* = 0.002	59 (67.0)	*p <* 0.001
Obstetric morbidity only (*n*, %)	18 (9.7)	16 (19.5)	*p* = 0.022	24 (27.3)	*p <* 0.001
Both (*n*, %)	1 (0.5)	5 (6.1)	*p* = 0.025	5 (5.7)	*p* = 0.029
**aPL profile (one determination)**					
Anti-β2GPI	-	-	-	45 (51.1)	-
LA	-	-	-	35 (39.7)	-
aCL	-	-	-	24 (27.3)	-
Double positive	-	-	-	17 (19.3)	-
Triple positive	-	-	-	1 (1.1)	-
**Associated AID (*n*, %)**	16 (8.6)	15 (18.3)	*p* = 0.026	11 (12.5)	*p* = 0.322
SLE	2 (1.1)	6 (7.3)	*p* = 0.017	2 (2.3)	*p* = 0.454
Plausible evolution to SLE	4 (2.2)	2/76 (2.6)	*p* = 0.828	4/86 (4.7)	*p* = 0.278
**Autoantibodies (*n*, %)**					
Antinuclear antibodies	34/142 (23.9)	37/73 (50.7)	*p <* 0.001	29/76 (38.2)	*p* = 0.026
**Cardiovascular risk factors (*n*, %)**					
Diabetes	9 (4.9(	3 (3.7)	*p* = 0.662	1 (1.1)	*p* = 0.160
Smoker	66 (35.7)	27 (32.9)	*p* = 0.664	19 (21.6)	*p* = 0.02
Arterial hypertension	23 (12.4)	17 (20.7)	*p* = 0.083	13 (14.8)	*p* = 0.594
Obesity	39 (21.1)	15 (18.3)	*p* = 0.601	16 (18.2)	*p* = 0.577
Dyslipidaemia	56 (30.3)	26 (31.7)	*p* = 0.814	26 (29.5)	*p* = 0.461
Hyperuricemia	5 (2.7)	6 (7.3)	*p* = 0.091	3 (3.4)	*p* = 0.707
**Other prothrombotic risk factors (*n*, %)**					
Hereditary thrombophilia	24 (13.0)	6 (7.3)	*p* = 0.214	12 (13.6)	*p* = 0.768
Oral contraceptive pill	76 (41.1)	19 (23.2)	*p <* 0.001	24 (27.3)	*p* = 0.002

*aCL* Anticardiolipin antibodies, *AID* Autoimmune disease, *aPL* Antiphospholipid antibodies, *APS* Antiphospholipid syndrome, *IQR* Interquartile range, *LA* Lupus anticoagulant, *SLE* Systemic lupus erythematosus

**Table 3:** “Non-criteria” clinical manifestations present in the seronegative antiphospholipid syndrome group

Clinical manifestation	
**Non-obstetric (*n*, %)**	***n*** **= 82**
Migraine	23 (28.0)
Brain MRI white matter lesions	21 (25.6)
Superficial vein thrombosis	19 (23.2)
Thrombocytopenia	17 (20.7)
*Livedo reticularis*	10 (12.2)
Valvular heart disease	8 (9.8)
Raynaud’s phenomenon	6 (7.3)
Seizures	6 (7.3)
Coombs’ positivity	5 (6.1)
Memory lapses	5 (6.1)
Transverse myelitis	3 (3.7)
Hemolytic anemia	3 (3.7)
Cognitive dysfunction	3 (3.7)
Pseudo-multiple sclerosis	3 (3.7)
Cardiac microvascular disease	3 (3.7)
Skin ulcers	2 (2.4)
APS nephropathy	2 (2.4)
Livedoid vasculopathy	1 (1.2)
**Obstetric**	***n*** **= 66**
Two spontaneous abortions <10 weeks	11 (16.7)
Late IUGR (>34 weeks)	5 (7.6)
Infertility	4 (6.1)
Premature birth between 34 and 37 weeks	3 (4.5)
Placental abruption	3 (4.5)
≥ 2 or more IVF failures	2 (3.0)
Puerperal preeclampsia	1 (1.5)
Late preeclampsia (>34 weeks)	1 (1.5)
Placental hematoma	1 (1.5)
Puerperal Thrombosis	1 (1.5)

*APS* Antiphospholipid syndrome, *IUGR* Intrauterine growth restriction, *IVF* In vitro fertilization, *MRI* Magnetic resonance imaging

### Comorbidities

Concomitant AID was more common in the SN-APS group (OR = 2.4, 95% CI 1.1–5.1, *p* = 0.026) than in the control group, with no significant difference between SP-aPL and control patients (*p* = 0.322) (Table 2). Both SN-APS and SP-aPL patients displayed less use of OCP compared with the control group (OR 0.3, 95% CI 0.15–0.5, *p* < 0.001, and OR 0.4, 95% CI 0.2–0.7, *p* = 0.002, respectively).

Positive ANA were more common in the SN-APS/SP-aPL groups (OR = 3.3, 95% CI 1.8–5.9, *p* < 0.001, and OR = 2.0, 95% CI 1.1–3.6, *p* 0.026, respectively) than in the control group, even when adjusting for the presence of associated AID (OR = 3.1, 95% CI 1.7–5.6, *p* < 0.001, and OR = 1.9, 95% CI 1.1–3.5, *p* = 0.033, respectively). Complement consumption was more common in the SN-APS group (OR = 5.4, 95% CI 1.4–21.2, *p* = 0.016), but this difference was not maintained when adjusting for the presence of associated AID (*p* = 0.067). No difference was found in the proportion of patients in which a progression towards a diagnosis of SLE was suspected between SN-APS (*p* = 0.828)/SP-aPL (*p* = 0.278) and the control group. Complete results are available in Table 2.

### Differences in clinical manifestations among groups

SN-APS and SP-aPL groups were associated with a higher frequency of obstetric manifestations in comparison with the control group (OR 3.0, 95% CI 1.5–6.0, *p* = 0.002, and OR 4.3, 95% CI 2.2–8.2, *p* < 0.001, respectively). Only one patient in the control group (0.5%) displayed both thrombotic and obstetric manifestations, a feature present in 6.1% and 5.7% of patients in the SN-APS (*p* = 0.025) and SP-aPL (*p* = 0.029) groups, respectively.

Concerning obstetric manifestations, no significant differences were found between SN-APS, SP-aPL and control patients. Additionally, no significant difference was observed in the recurrence of obstetric events and number of abortions between SN-APS/SP-aPL patients and the control group. Complete results are available in Table 4.

**Table 4:** Patient clinical manifestations and treatment

Clinical manifestations	Control group (***n*** = 185)	Seronegative APS (***n*** = 82)	***P***-value (SN-APS vs. controls)	Single-positive aPL (***n*** = 88)	***P***-value (SP-APS vs. controls)
**Thrombosis**	***N*** **= 167**	***n*** **= 66**		***n*** **= 64**	
**Number of thrombotic events (median, IQR)**	1 [1–1]	1.5 [1–2]	*p<*0.001	1 [1–2]	*p*=0.004
Arterial thrombosis only (*n*, %)	77 (46.1)	24 (36.4)	*p*=0.268	14 (21.9)	*p*=0.007
Venous thrombosis only (*n*, %)	79 (47.3)	33 (50.0)	*p*=0.698	43 (67.2)	*p*=0.208
Both arterial and venous thrombosis (*n*, %)	11 (6.6)	9 (13.6)	*p*=0.156	7 (10.9)	*p*=0.533
Stroke (*n*, %)	48 (28.7)	22 (33.3)	*p*=0.894	9 (14.1)	*p*=0.008
TIA (*n*, %)	9 (5.4)	8 (12.1)	*p*=0.408	3 (4.7)	*p*=0.581
Limb ischemia (*n*, %)	6 (3.6)	6 (9.1)	*p*=0.783	3 (4.7)	*p*=0.699
Acute myocardial infarction (*n*, %)	21 (12.6)	6 (9.1)	*p*=0.189	9 (14.1)	*p*=0.669
Pulmonary embolism (*n*, %)	27 (16.2)	18 (27.3)	*p*=0.262	18 (28.1)	*p*=0.136
DVT (*n*, %)	56 (33.5)	28 (42.4)	*p*=0.157	21 (32.8)	*p*=0.656
Cerebral vein thrombosis (*n*, %)	19 (11.4)	6 (10.6)	*p*=0.228	14 (21.9)	*p*=0.045
Retinal vessels thrombosis (*n*, %)	3 (1.8)	2 (3.0)	*p*=0.349	2 (3.1)	*p*=0.827
**Obstetric**	***N*** **= 19**	***n*** **= 21**		***n*** **= 29**	
**Number of abortions (median, IQR)**	3.0 [1–5]	3 [1.5–4]	*p* = 0.473	3 [1–4]	*p* = 0.384
More than three abortions <10 weeks (*n,* %)	10 (52.6)	5 (23.8)	*p* = 0.065	15 (48.3)	*p* = 0.951
Miscarriage >10 weeks (*n*, %)	8 (42.1)	14 (66.7)	*p* = 0.123	11 (37.9)	*p* = 0.773
Prematurity <34 weeks (*n*, %)	3 (15.8)	5 (23.8)	*p* = 0.529	3 (10.3)	*p* = 0.579
Placental ischemia (*n*, %)	5 (26.3)	12 (57.1)	*p* = 0.054	8 (27.6)	*p* = 0.923
**Treatment**					
**Thrombosis patients**	***n*** **= 167**	***n*** **= 66**		***n*** **= 64**	
Indefinite anticoagulation (*n*, %)	54 (32.3)	40 (60.6)	*p <* 0.001	33 (51.6)	*p* = 0.008
Vitamin K antagonist (*n*, %)	29 (17.4)	28 (42.4)	*p <* 0.001	28 (43.8)	*p <* 0.001
Direct oral anticoagulant (*n*, %)	25 (15.0)	12 (18.2)	*p =* 0.546	5 (7.8)	*p =* 0.155
Antiplatelet therapy (*n*, %)	68 (40.7)	23 (34.8)	*p* = 0.408	13 (20.3)	*p* = 0.004
Hydroxychloroquine (*n*, %)	1 (0.6)	5 (7.6)	*p* = 0.018	5 (7.8)	*p* = 0.017
Anticoagulation duration [median (months), IQR]	20.5 [11–73.8]	74.5 [23.3–114.8]	*p <* 0.001	61 [14–113]	*p* = 0.013
**Obstetric patients (during pregnancy) (*n*, %)**	***n*** **= 18**	***n*** **= 21**		***n*** **= 29**	
Any treatment	13 (72.2)	11 (52.4)	*p* = 0.209	16 (55.2)	*p* = 0.246
Aspirin monotherapy	2 (11.1)	0 (0)	*p* = 0.998	3 (10.3)	*p* = 0.934
LMWH/aspirin combination	11 (61.1)	10 (47.6)	*p* = 0.401	13 (44.8)	*p* = 0.280
Hydroxychloroquine	6 (33.3)	3 (14.3)	*p* = 0.169	3 (10.3)	*p* = 0.063

*TIA* Transitory ischemic attack, *aPL* Antiphospholipid antibodies, *APS* Antiphospholipid syndrome, *IQR* Interquartile range, *LMWH* Low-molecular-weight heparin

No significant differences were found between SN-APS/SP-aPL and the control groups in the frequency of venous thrombosis (*p* = 0.698 and *p* = 0.208, respectively). On the other hand, the SP-aPL group displayed significantly less arterial thrombosis than the control group (OR = 0.4, 95% CI 0.2–0.8, *p* = 0.007), with no significant difference between SN-APS and control patients (*p* = 0.711). Concerning specific thrombotic manifestations, SP-aPL patients displayed significantly less frequency of stroke (OR = 0.3, 95% CI 0.1–0.7, *p* = 0.008) but more frequent cerebral vein thrombosis (OR = 2.2, 95% CI 1.01–4.7, *p* = 0.045) than patients from the control group. No significant differences were found in the remaining clinical manifestations between SN-APS/SP-aPL and the control group. Complete results are available in Table 4.

### Thrombosis recurrence

**Fig. 1:**
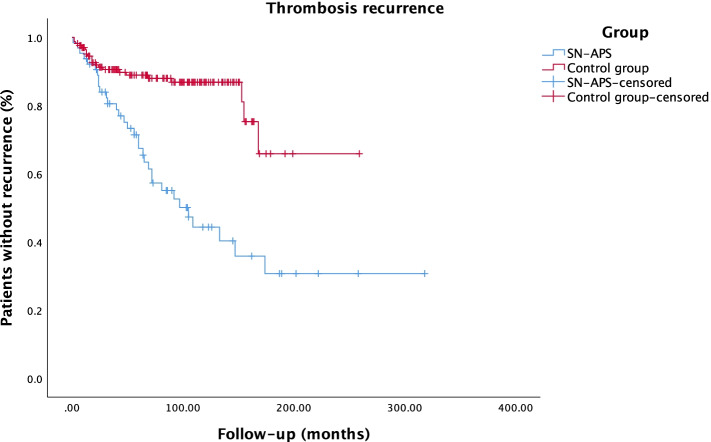
Kaplan-Meier curve of overall survival (absence of thrombosis recurrence) of seronegative antiphospholipid syndrome and controls

**Fig. 2:**
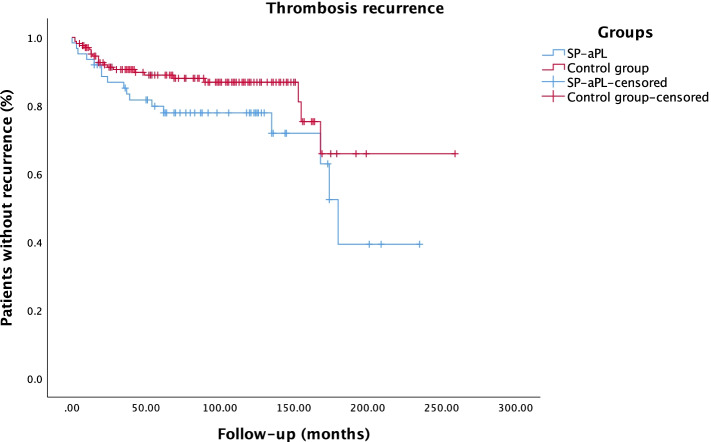
Kaplan-Meier curve of overall survival (absence of thrombosis recurrence) of single-positive aPL patients and controls

Regarding thrombosis recurrence, in a Cox regression model, SN-APS associated with significantly higher chances of recurrence than the control group (HR = 3.8, 95% CI = 2.2–6.5, *p* < 0.001) (Fig. 1). Similar results were observed after adjusting for the presence of hereditary thrombophilia (HR 3.8, 95% CI 2.1–6.6, *p* < 0.001), associated SLE (HR 3.8, 95% CI 2.2–6.6, *p* < 0.001) or OCP (HR 5.7, 95% CI 2.6–12.6, *p* < 0.001). In the case of SP-aPL group, we observed a non-significant trend for a higher chance of recurrence both in unadjusted analysis (HR 1.8, 95% CI 0.9–3.4, *p* = 0.078) (Fig. 2) and after adjusting for the presence of hereditary thrombophilia (HR 1.8, 95% CI 0.9–3.5, *p* = 0.078), associated SLE (OR 1.9, 95% CI 0.98–3.5, *p* = 0.06) or OCP (HR 2.3, 95% CI 0.97–5.3, *p* = 0.057). The mean number of thrombotic events was higher in both SN-APS and SP-aPL groups in comparison with the control group (regression coefficient = 0.6, 95% CI 0.4–0.7, *p* < 0.001, and regression coefficient = 0.2, 95% CI 0.1–0.3, *p* = 0.004, respectively). No patient in the control group displayed recurrence under anticoagulation, a feature present in 10.6% and 4.7% of patients in the SN-APS and SP-aPL groups, respectively.

When assessing SN-APS patients among themselves, no particular non-criteria manifestation was specifically associated with thrombosis recurrence. When selecting SN-APS patients with specific non-criteria features and comparing them with the control group, various manifestations were statistically associated with recurrence, albeit the OR’s displayed wide confidence intervals, hinting a low estimate precision due to small sample size: thrombocytopenia (OR 9.9, 95% CI 3.2–30.5, *p* < 0.001), brain white matter lesions (OR 11.3, 95% CI 4.0–31.8, *p* < 0.001), migraine (OR 9.9, 95% CI 3.6–26.9, *p* < 0.001), superficial vein thrombosis (OR 3.3, 95% CI 1.1–9.7, *p* = 0.03), and seizures (OR 9.9, 95% CI 1.6–62.5, *p* = 0.015).

### Differences in treatment among groups

In patients with thrombosis, indefinite anticoagulation was more common both in SN-APS and SP-aPL groups comparing with the control group (OR 3.2, 95% CI 1.8–5.8, *p* < 0.001, and OR 2.2, 95% CI 1.2–4.0, *p* = 0.008, respectively). Additionally, a longer global duration of anticoagulation was observed in SN-APS (regression coefficient = 46.4; 95% CI 33.0–55.1, *p* < 0.001) and SP-aPL patients (regression coefficient = 20.8; 95% CI 4.7–36.9, *p* = 0.012) in comparison with the control group. In anticoagulated patients, anticoagulation with a VKA (instead of a DOAC) was more common in the SN-APS/SP-aPL groups than in the control group (OR = 3.5, 95% CI 1.9–6.6, *p* < 0.001, and OR = 3.7, 95% CI 2.0–7.0, *p* < 0.001, respectively). In patients with obstetric manifestations, no significant difference was observed in pregnancy treatment between SN-APS/SP-aPL patients and the control group. Complete results are available in Table 4.

## Discussion

Even though various non-criteria manifestations are being considered for inclusion in the new Classification Criteria for APS currently under development [2], there are few studies specifically tackling the relevance of these features [7, 20]. To our knowledge, this is the first study to address the impact of non-criteria APS manifestations as a whole in seronegative patients with APS criteria manifestations. The additional inclusion of a group of patients with single aPL positivity provides further data on a controversial domain for which there is limited evidence available.

In our series, patients with non-criteria manifestations (SN-APS) displayed a higher frequency of obstetric morbidity and concurrent obstetric and thrombotic manifestations in comparison with controls. Additionally, ANA positivity was more prevalent even when taking into account the presence of other AID, hinting a possible role as a marker of autoimmunity in these patients. When focusing on patients with thrombosis, although the type of events did not differ from controls, a significantly higher number of events and thrombosis recurrence was observed in patients with non-criteria manifestations, a deed still sustained even after adjusting for various relevant confounders (i.e., contraceptive pill use, associated SLE or hereditary thrombophilia, follow-up duration). A previous work described similar prevalence of thrombosis recurrence between patients with SN-APS (defined as clinical manifestations of APS but testing negative for criteria aPL plus the presence of at least two non-criteria manifestations) and definite APS patients [6]. Our data sheds new light on the potential impact of non-criteria manifestations on the prognosis of these patients; it is curious to notice that this information might already influence daily clinical practice, as these patients in our cohort were also more frequently under indefinite anticoagulation, displayed longer anticoagulation duration, and a higher use of VKA instead of DOAC.

Regarding the impact of specific non-criteria manifestations on thrombosis recurrence, the significance of the observed associations is undermined by the small sample size possibly leading to estimates of low precision and misleading high magnitude. Nevertheless, there is already some evidence in the literature portraying a possible role of these manifestations. In the case of thrombocytopenia, although previous data provided conflicting results, most publications increasingly support a potential impact of this feature in APS prognosis. While two studies found no significant difference in thrombosis recurrence among APS patients with and without thrombocytopenia [21, 22], a study of 138 patients with aPL positivity and thrombocytopenia (i.e., fulfilling laboratory but not clinical criteria of APS) described a five times higher risk of future thrombosis in these patients compared with those with normal platelet counts [23], and another publication described that, in aPL-positive patients, those with a low platelet count developed thrombosis more frequently than those without [24]. Moreover, a study found significantly higher adjusted Global APS Score (aGAPSS) values in APS patients with thrombocytopenia when compared to patients without non-criteria manifestations [25]. Concerning *livedo reticularis*, a previous work reported an increased frequency of this feature in patients with arterial events and decreased frequency in those with venous events [8, 26]. In respect to superficial vein thrombosis, in a prospective study of patients with SLE and/or aPL, its presence carried a hazard ratio of 7.45 for the occurrence of thromboembolic events, suggesting a possible prognostic significance [27]. Relative to brain white matter lesions, APS patients frequently display abnormalities on neuroimaging studies, most commonly focal subcortical white matter areas of signal hyperintensity [28, 29]. It is not always clear whether these lesions represent ischemia or inflammation [28, 29], with hints pointing towards an APS diagnosis including smaller lesions on MRI, frequently located in the subcortical area, with stability over time and possible improvement with anticoagulation therapy [30]. The evidence of a potential impact of these lesions in patients’ prognosis gains relevance as their significance is still debated in APS. There is a controversial possibility linking white matter lesions with the presence cognitive impairment, but while some MRI studies in APS patients with neurological symptoms display high frequency of infarcts, others focusing specifically on cognition did not demonstrate an increased number of infarcts in APS patients with cognitive deficits comparing with controls [28].

Regarding SP-aPL patients, in a similar fashion to SN-APS, they also displayed a higher frequency of obstetric criteria manifestations and concurrent obstetric and thrombotic manifestations in comparison with controls. Focusing on thrombosis, even though the mean number of events was higher in SP-aPL, recurrence was not significantly different from controls. This is in line with the notion that one single aPL positive determination is not associated with increased recurrent thrombosis, as stated in the previous systematic review reporting, in these patients, a similar recurrence rate to the general population [14].

Lastly, the comparison of the characteristics and treatment of obstetric patients between SN-APS/SP-aPL patients and the control revealed no significant differences, but the small sample undermines the extraction of significant and generalizable conclusions.

This work encompasses significant strengths but also limitations. First, as these patients did not have definite diagnosis, they were managed according to their physician’s judgment and not respecting a predefined protocol, leading to potential disparities in their treatment. The treatment options might also reflect specific management approaches of the studied centers and not be easily comparable with other institutions. Additionally, the retrospective design and the fact that both SN-APS and SP-aPL groups include quite heterogeneous individuals (i.e., patients with varied non-criteria manifestations and single positivity of different antibodies) weaken the extrapolation of obtained results. Nevertheless, considering the rarity of the entity and the difficulties in identifying these patients, a retrospective design is a feasible first approach to gather initial data in a domain with practically no available evidence. This is also valid regarding the different non-criteria manifestations. The report of the *14th International Congress on Antiphospholipid Antibodies Technical Task Force on APS Clinical Features* reviewed the literature devoted to some of these non-criteria manifestations, and the sparsity of data regarding their impact was clear. Even in the new criteria under development [2], part of the decision to include or not these non-criteria manifestations in the preliminary criteria included a share of eminence-based assessment, as experts classified clinical scenarios with these features as “highly likely” or “equivocal or unlikely” APS. Therefore, the fact that our work included patients from two different centers, with clearly defined manifestations and inclusion criteria, and an adjustment for relevant confounders when comparing with the control group, constitutes a pertinent, though initial, effort to provide data in a domain with limited and heterogenous guiding evidence.

These results carry clinical implications, suggesting that the presence of non-criteria manifestations negatively affects the prognosis of SN-APS. This could imply a potential need for a more thorough follow-up and aggressive management of these patients, with earlier and prolonged anticoagulation. Conversely, the presence of only one single aPL positive determination does not seem to dictate increased risk of recurrent thrombosis, serving as a reinforcement to the current practice of managing these patients in a similar fashion to the general population. However, confirmation of these results should be obtained in future prospective, ideally multicenter studies (considering the scarcity of these patients), ideally focusing on specific non-criteria manifestations.

## Conclusion

SN-APS patients displayed more thrombosis recurrence, indefinite anticoagulation, use of VKA (instead of DOAC), and longer anticoagulation duration than controls without non-criteria manifestations. SP-aPL patients did not display significantly higher thrombosis recurrence in comparison with controls. The presence of non-criteria manifestations in patients with thrombosis and negative aPL may negatively impact the clinical course of these patients and confer a poorer prognosis.

## Data Availability

The datasets used and/or analyzed during the current study are available from the corresponding author on reasonable request.
